# The probability of wing damage in the dragonfly *Sympetrum vulgatum* (Anisoptera: Libellulidae): a field study

**DOI:** 10.1242/bio.027078

**Published:** 2017-07-27

**Authors:** Hamed Rajabi, Veronica Schroeter, Shahab Eshghi, Stanislav N. Gorb

**Affiliations:** 1 Institute of Zoology, Functional Morphology and Biomechanics, Kiel University, D-24118 Kiel, Germany; 2 Young Researchers and Elite Club, Lahijan Branch, Islamic Azad University, Lahijan, Iran

**Keywords:** Dragonfly, Wing, Damage, Adaptation, Wear, Collision

## Abstract

Dragonfly wings resist millions of cycles of dynamic loading in their lifespan. During their operation, the wings are subjected to relatively high mechanical stresses. They further experience accidental collisions which result from the insects' daily activities, such as foraging, mating and fighting with other individuals. All these factors may lead to irreversible wing damage. Here, for the first time, we collected qualitative and quantitative data to systematically investigate the occurrence of damage in dragonfly wings in nature. The results obtained from the analysis of 119 wings from >30 individual *Sympetrum vulgatum* (Anisoptera: Libellulidae), collected at the second half of their flight period, indicate a high risk of damage in both fore- and hindwings. Statistical analyses show no significant difference between the extent of damage in fore- and hindwings, or between male and female dragonflies. However, we observe a considerable difference in the probability of damage in different wing regions. The wing damage is found to mainly result from two failure modes: wear and fracture.

## Abstract (precis)

**Summary:** This study provides the first qualitative and quantitative data on the occurrence of damage in the wings of the dragonfly *Sympetrum vulgatum* in nature. This article has an associated First Person interview with the first author of the paper as part of the supplementary information.

## INTRODUCTION

Dragonflies are one of the most impressive fliers among flying insects. Their fascinating flight performance is known to be strongly influenced by the unique material composition and complex structural design of their wings (Rajabi et al., 2016a,b, 2017a; Wootton and Newman, 2008). The flight capabilities of dragonflies offer significant advantages in terms of foraging, escaping from predators, mating, dispersing and finding new habitats. However, the wings have extremely light-weight structures and, similar to wings of other flying insects, may experience structural damage during their lifespan (Wootton, 1992). Due to the lack of healing, such damage is irreversible (Hayes and Wall, 1999), and is believed to reduce the survival of the insects (Combes et al., 2010). Therefore, it is plausible to expect that the wings must be adapted to mitigate damage caused by excessive mechanical stresses. This hypothesis is supported by a few recent studies showing that insect wings exhibit several biomechanical adaptations to prevent failure (Mountcastle and Combes, 2014; Rajabi et al., 2016c).

To the best of the authors' knowledge, there are no data in the literature regarding the occurrence of damage in dragonfly wings in nature. Independent from the causes of the wing damage and its influence on the flight performance of dragonflies, such information is believed to provide valuable insights into damage-tolerance mechanisms in dragonfly wings. Therefore, in this study, we focus on the investigation of wing damage in the dragonfly *Sympetrum vulgatum* in nature (and not under laboratory conditions). Our aim is to answer the following questions:

- How frequently does damage occur in dragonfly wings?
- Which parts of the wings are more susceptible to damage?
- Does the extent of damage differ between fore- and hindwings or between the wings of male and female insects?
- Which failure mechanisms are more likely to occur in the wings under natural conditions?

## RESULTS AND DISCUSSION

Damage was found in almost 76.2% of the forewings and 78.6% of the hindwings investigated in this study. This indicates a very high risk of wing damage in adult dragonflies in nature. Such damage may result from physical interactions of the wings with surrounding objects (mostly vegetation), bodies of other conspecific or heterospecific dragonfly individuals (Foster and Cartar, 2011), and predators during their attacks. Although most of the damage resulted in the loss of <10% of wing area, we observed a few severe cases of wing area loss ≤75%. Fig. S1 illustrates a few cases of minor, moderate and severe wing damage (the images of all investigated wings are available online at https://doi.org/10.6084/m9.figshare.5104453.v1).

Hindwings in dragonflies have a larger area than forewings (∼1.3 times). That is why, when comparing damaged areas, we observed a relatively greater area loss in hindwings than in forewings. However, statistical analysis showed no significant difference between the ratios of the wing damaged area to the whole wing area in fore- and hindwings (Mann–Whitney *U*-test, *P*=0.211, *n*=92) (Fig. S2A). Therefore, when considering wing area loss as a fraction of whole wing area, both wings appear to have almost the same risk of damage in the second half of the flight season (details regarding the insect flight period can be found in Gorb and Pavlyuk, 1993). Fig. S3A,B show two histograms representing the number of damaged fore- and hindwings exhibiting different percentages of damaged area to whole wing area.

No correlation was found between the ratio of damaged area to the wing area and dragonfly sex (Mann–Whitney *U*-test, *P*=0.819, *n*=92) (Fig. S2B). This result indicates that the wings of a female dragonfly may experience as much damage as those of a male one. This finding was initially quite surprising, as we expected to find more damage in the wings of male dragonflies due to their territorial activities. Males are known to usually fight with each other when defending their territory or seeking mates, factors that might increase the risk of damage to their wings. However, one should also take into account that female dragonflies may experience severe sexual harassment (Fincke, 2004); at the mating sites, males try to seize females and often grasp their wings (Movie 1). Additionally, strong interactions occur between female *Sympetrum* dragonflies at foraging sites (Gorb, 1994). In a recent study, we have shown the occurrence of multiple physical contacts between the body of a male dragonfly and wings of a female dragonfly when forming a tandem pair (Rajabi et al., 2016c). Furthermore, females and males fly in tandem over the water surface while females are laying eggs. This flight behaviour may remarkably increase the accidental collisions with vegetation in both sexes. These factors are presumably responsible for the damage in wings of female dragonflies as well as those of male dragonflies. The number of damaged wings, and percentage of damaged area to wing area, is shown in Fig. S3C,D for male and female dragonflies, respectively.

**Fig. 1.**
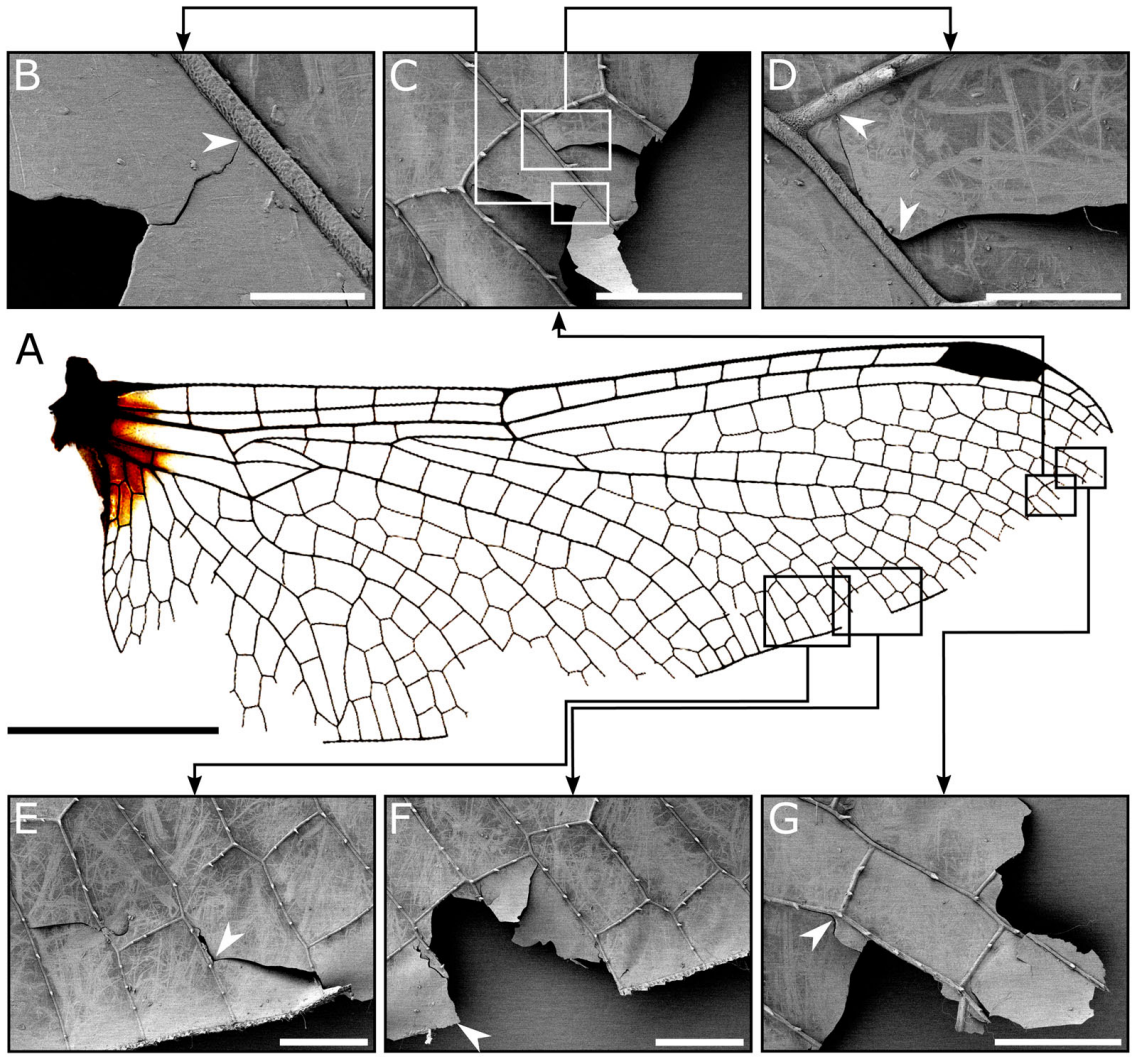
**The** **damaged parts of a hindwing sample.** (A) Hindwing of the dragonfly *S. vulgatum*. (B-G) SEM images of the damaged regions of the dragonfly hindwing. Numerous scratches can be found on the wing surface. (D-F) The occurrence of wear is obvious in many wing regions. (E,F) The wing veins may be able to stop or deflect a propagating crack (arrowheads). Scale bars: 0.5 cm in A; 50 µm in B; 500 µm in C, E, F and G; 100 µm in D.

Fig. 1 represents a set of scanning electron microscopy (SEM) images showing the damaged parts of a hindwing sample. As seen in Fig. 1D-F, numerous scratches can be found on the wing surface, as previously described for other odonate species (Gorb et al., 2000, 2009). The considerably high number of scratches near the wing margin has resulted in complete removal of the crystalline wax wing coverage in this region (Fig. 1E,F). This finding supports the occurrence of abrasive wear in dragonfly wings. It can be seen that the excessive wear of the wing margin in the left-hand side of Fig. 1F led to the removal of the wing trailing edge (arrowhead). The loss of the wing material due to wear is supposed to increase the probability of crack formation.

Fig. 1E illustrates the presence of an edge crack, indicating the tearing of the wing margin. It is likely that the crack has been initiated at the edge. After breaking the first cross vein, it has reached the second one. At this point, the crack has deflected and continued to grow parallel to the vein (arrowhead). The stop and deflection of a propagating crack can be found in several cases (Fig. 1B-E,G). In such cases, a deflected crack has to propagate a longer distance than one extending along a straight line, before it advances to a neighbouring cell. This results in dissipation of higher amount of energy, and therefore delays the wing catastrophic failure. These observations provide further evidence of the crucial role of veins in toughening the wing structure (Dirks and Taylor, 2012; Rajabi et al., 2015, 2017b).

**Fig. 2.**
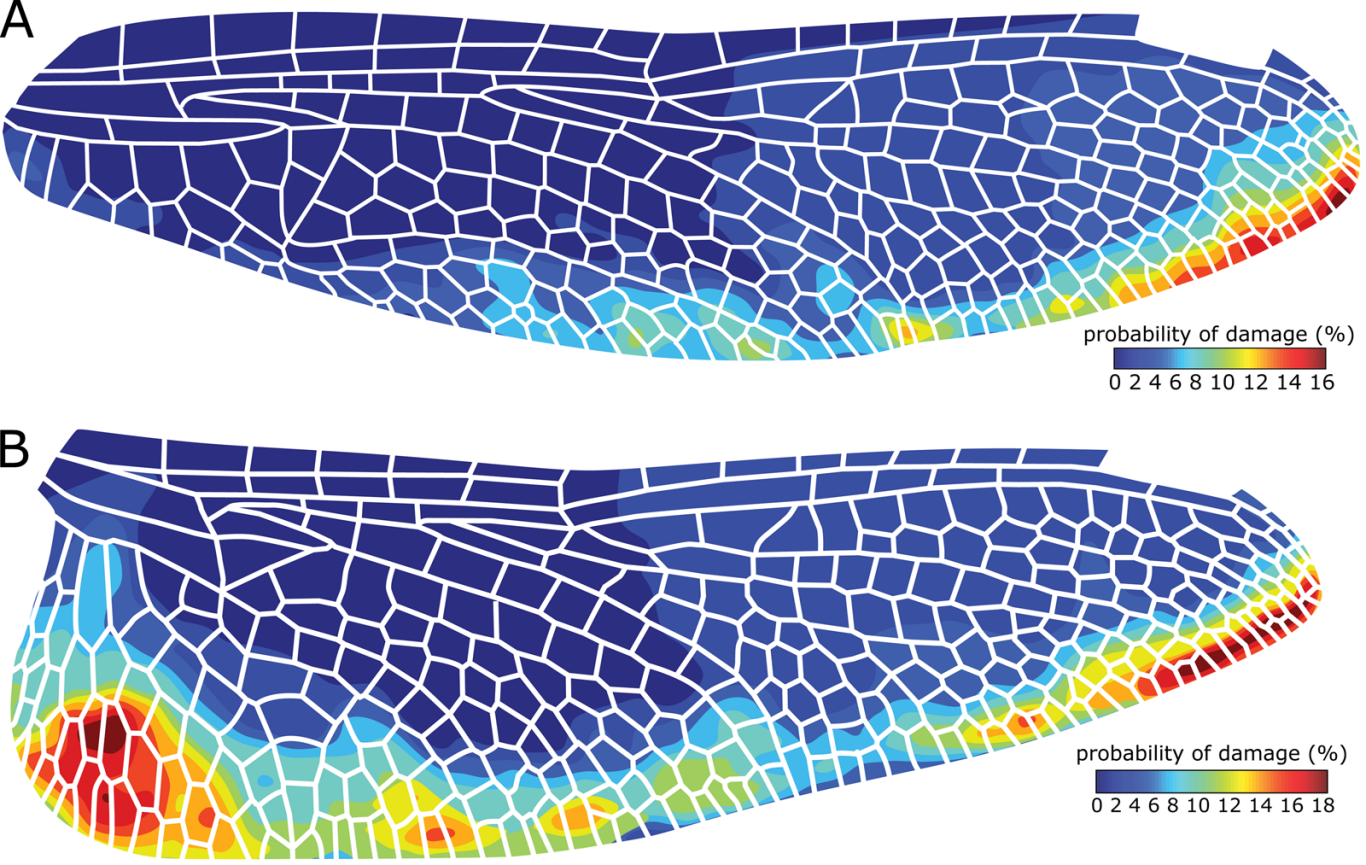
**Probability of damage in the fore- and hindwing of the dragonfly *S. vulgatum*.** In the (A) forewing and (B) hindwing, the wing cells near the trailing edge seem to be more susceptible to damage. The probability of damage is much higher in the posterior-distal part of both wing pairs and at the broad base of the hindwing.

Fig. 2 illustrates the probability of damage in different wing regions. As can be seen here, in both wings, the posterior part, and especially the regions near the trailing edge, are more susceptible to damage. The cells located at the posterior-distal part of the forewing were found to have damage in 16% of the analysed specimens. The probability of damage in the cells from a similar part in the hindwing is slightly higher and can reach up to 18%. In addition to this wing region, the posterior-proximal part of the broad-based hindwings seems to be another critical zone, showing a relatively high risk of damage. These observations are particularly interesting in the light of the fact that the loads acting on the wings during flight and those due to collisions are likely to induce large bending moments and, therefore, high mechanical stresses near the wing root (Ennos, 1989). The aerodynamic forces are also known to be greater at the leading edge, in comparison to the trailing edge (Ellington et al., 1996). Thus, we expected to find more damage at the base and leading edge than in other wing regions.

A possible reason for the observed spatial variation of damage may be variable experience of mechanical stress by different wing regions, resulting from the nature of physical interaction between the wings and objects in the environment. A more likely reason, however, may be the nonuniform cuticle thickness distribution of the wings. The veins and membrane located in the posterior part of fore- and hindwings have much smaller thickness compared to those in the anterior part (Jongerius and Lentink, 2010). The relatively small thickness of the wing in the posterior part is known to contribute to the aerodynamic lift generation by improving the flexibility of this wing region (Mountcastle and Combes, 2013). However, on the other hand, this may increase the likelihood of wear-related damage, as we showed here.

Wing damage may remarkably shorten the lifespan of insects by imposing additional costs on their flight performance (Combes et al., 2010), reducing the prey capture success (Combes et al., 2010) and increasing the risk of being captured by predators (Rodd et al., 1980). The results of the present study suggest that some parts of the wings may be adapted to be more damage-tolerant than others. The information on the biomechanical adaptations and mechanisms contributing to the high resistance of these wing regions is likely to help researchers to design more durable biomimetic structures. Future research is planned to monitor the formation and temporal progression of damage in dragonfly wings under natural conditions.

## MATERIALS AND METHODS

The *S. vulgatum* dragonflies were caught near Nyzhny Mlyny (Poltava Province, Ukraine) during mid-September 2016. The insects were anaesthetized with chloroform vapour and air-dried at room temperature. In the laboratory, the wings were carefully separated from the bodies using a sharp razor blade and scanned under an optical surface scanner microscope (VR-3000 Series, Keyence, Osaka, Japan). These procedures comply with ethical guidelines at Kiel University. The obtained digital images were examined to determine whether individual wings contained any damage. Among 119 investigated wings (63 forewings and 56 hindwings from >30 dragonflies), 48 forewings and 44 hindwings were found to have damage in different levels.

To measure the area loss, an image of an intact wing was selected and overlaid onto the image of each individual damaged wing. The intact wing image was then resized and reoriented to match the dimensions of the damaged wing under investigation. Through this method, we were able to identify the missing edges of our damaged wings, in reference to the intact wing. ImageJ image processing software (https://imagej.nih.gov/ij/) was used to mark and then measure the area of the damaged parts of the wings, as a fraction of the whole wing area.

A custom MATLAB script (available on request) was employed to calculate the probability of damage in different wing regions. To this end, we first had to introduce an ‘idealized’ wing. An intact wing image was selected and scaled, in both spanwise and chordwise directions, to achieve a length and width equal to the mean length and width of the collected wings, respectively (forewing length 28.53±0.41 mm; forewing width 6.97±0.29 mm; hindwing length 27.52±0.51 mm; hindwing width 9.89±0.31 mm). This process was performed separately for both fore- and hindwings, allowing us to introduce an idealized forewing and an idealized hindwing. In this step, the wing images with marked damaged parts were imported into the MATLAB script. The individual wing images were then automatically oriented and resized to match the dimensions of their corresponding idealized wings. This resulted in a set of wing images, of which all had the same orientation and dimensions. The coordinates of the pixels in the marked regions in each single image were collected with respect to the most proximal point at the wing base, as a fixed reference point, and stored in a cell array. Finally, the number of repetitions of the stored pixels with the same coordinates was calculated and divided by the number of the analysed images.

The data from measurements were tested for significant differences. Since the data sets were unpaired and non-normally distributed, the nonparametric Mann–Whitney *U*-test was utilized.

We further employed a scanning electron microscope (Hitachi S-4800, Hitachi High-Technologies, Tokyo, Japan) to identify the type of damage in the insect wings. The wing samples used in the microscopic studies were mounted on holders using carbon Leit-Tabs (Plano GmbH, Wetzlar, Germany). An EM SCD 500 high-vacuum sputter coater (Leica Microsystems, Mannheim, Germany) was utilized to coat the samples with a thin layer (8-9 nm) of gold-palladium. The specimens were imaged at an accelerating voltage of 3 kV.
